# Chemokines in Cartilage Regeneration and Degradation: New Insights

**DOI:** 10.3390/ijms25010381

**Published:** 2023-12-27

**Authors:** Bouchra Edderkaoui

**Affiliations:** 1 Musculoskeletal Disease Center, Research Service, VA Loma Linda Healthcare Systems, Loma Linda, CA 92357, USA; bouchra.edderkaoui@va.gov; 2 Department of Medicine, Loma Linda University, Loma Linda, CA 92354, USA

**Keywords:** chemokines, chemokine receptors, cartilage formation, cartilage degeneration

## Abstract

Cartilage plays a crucial role in the human body by forming long bones during development and growth to bear loads on joints and intervertebral discs. However, the increasing prevalence of cartilage degenerative disorders is a growing public health concern, especially due to the poor innate regenerative capacity of cartilage. Chondrocytes are a source of several inflammatory mediators that play vital roles in the pathogenesis of cartilage disorders. Among these mediators, chemokines have been explored as potential contributors to cartilage degeneration and regeneration. Our review focuses on the progress made during the last ten years in identifying the regulators and roles of chemokines and their receptors in different mechanisms related to chondrocytes and cartilage. Recent findings have demonstrated that chemokines influence cartilage both positively and negatively. Their induction and involvement in either process depends on the local molecular environment and is both site- and time-dependent. One of the challenges in defining the role of chemokines in cartilage pathology or regeneration is the apparent redundancy in the interaction of chemokines with their receptors. Hence, it is crucial to determine, for each situation, whether targeting specific chemokines or their receptors will help in developing effective therapeutic strategies for cartilage repair.

## 1. Introduction

**Cartilage:** The cartilage is a highly specialized connective tissue that is avascular, aneural, and alymphatic. The main components of cartilage are the extracellular matrix (ECM) and chondrocytes. There are three types of cartilage tissue: hyaline cartilage (costal, nasal septum, and articular cartilage of the joints), fibrous cartilage (annulus fibrosus of the spine discs, eustachian/auditory tube, and joints), and elastic cartilage (auricle of the ear and epiglottis of the throat). Articular cartilage in the joints receives nutrients through diffusion from the synovial fluid, which is rich in proteins derived from the blood plasma and joint tissues (hyaluronic acid and proteoglycan 4) [1]. During skeletal development, the hyaline cartilage develops first in the embryos and later transforms into other types of cartilage and bone tissues. Unlike other cartilage tissues, adult fibrous cartilage mainly contains type I collagen. It is found in the articular lips, discs, menisci, and intervertebral discs and serves as a transitional tissue between the dense connective tissue (tendon) and hyaline cartilage.

The increasing prevalence of degenerative cartilage disorders in the joints of young and adult patients is a growing public health concern, especially because of the poor regenerative capacity of the cartilage. Therefore, further investigations are required to reduce cartilage degeneration and improve its regenerative ability. Chondrocytes are a source of several inflammatory mediators that play critical roles in the pathogenesis of osteoarthritis (OA) [2]. Of these mediators, chemokines have been studied for their role in cartilage degeneration and as potential targets for cartilage regeneration, as they are secreted from both stimulated and unstimulated chondrocytes as well as from inflammatory cells that migrate to injured or infected cartilage.

**Chemokines:** These are small chemotactic cytokines ranging in size from 8 to 20 kDa and sharing a basic structure stabilized by disulfide bonds between the conserved cysteine residues. Based on the pattern of cysteine residues near the N-terminus, chemokines can be divided into four subfamilies [3]: (1) the CC subfamily, which includes the beta chemokines and has the two cysteine residues adjacent to each other; (2) the CXC subfamily, which consists of alpha chemokines that have the two conserved cysteine residues separated by an intervening amino acid; (3) the C subfamily, which has one cysteine residue at the N-terminus of the protein; and (4) the CX3C subfamily, with only one representative, CX3CL1, also known as neurotactin in mice and fractalkine in humans, in which the two cysteine residues are separated by three amino acids [4]. Chemokines are secreted by various cell types. Their name derives from their ability to induce directed chemotaxis in nearby cells by binding to G-protein-coupled receptors (GPCRs), which have a characteristic of seven-transmembrane structure. Some chemokines are considered pro-inflammatory, and they are induced during immune responses to attract cells of the immune system to the site of infection or injury; however, others are considered homeostatic and are involved in controlling cell migration under normal physiological conditions. Chemokines are involved in cell maturation, differentiation, infection, autoimmunity, cancer, and other conditions associated with the immune system. Nevertheless, the mechanisms modulating their expression and function or their roles in regulating chondrocyte proliferation, differentiation, and senescence remain unclear. This review focuses on the progress made over the last ten years by compiling and analyzing published data related to the expression, roles, and mechanisms of action of chemokines in cartilage development and disease.

## 2. Method

The peer-reviewed literature for each chemokine published during the last ten years in the PubMed database was searched using the following keywords: Chemokine name AND Cartilage. We excluded publications in which cartilage was only mentioned in the introduction and/or discussion or cartilage tissue/chondrocytes were not used in the study, or if the study was a confirmation of parts of previous findings, either in the same model or different models. Relevant articles selected based on research advances related to the mechanisms that regulate the expression and function of chemokines/receptors, along with their role in chondrogenesis or cartilage pathology, are summarized and discussed below.

Information from old publications has been included to provide background information on specific chemokines and their receptors and to add context to new findings.

## 3. Results and Discussion

### 3.1. Monocyte Chemotactic Proteins (MCPs)

#### 3.1.1. MCP-1

MCP-1 is also known as chemokine (C-C motif) ligand 2 (CCL2), and it is one of the most widely studied chemokines. It is expressed in synovial cells [5], chondrocytes [2], osteoblasts [6], and other cell types [7]. High levels of CCL2 in serum and synovial fluid positively correlate with the severity of knee OA symptoms [5,8]. CCL2 is one of the strongest intraoperative predictors of severe cartilage lesions independent of associated pathologies [9]. In a comparative study, CCL2 levels were found to be higher in the serum of patients with both knee OA and hip OA than in normal controls [10]. However, the levels of most of the other chemokines were differentially affected between hip OA and knee OA. Furthermore, Raghu et al. [5] demonstrated that mice deficient in *CCL2* or its receptor, *CCR2*, were protected from OA development after medial meniscus destabilization (MMD), with a concomitant reduction in monocyte and macrophage recruitment to the injured knees (Table 1). However, a previous study by Poo et al. [11] reported a substantial increase and prolonged arthritic disease in *CCR2*-KO mice infected with chikungunya virus compared to wild-type mice, as evidenced by cartilage damage in the knee joints of *CCR2*-KO mice (Table 1), while no evidence of cartilage degeneration was observed in the knee joints of wild-type mice. In *CCR2*-KO mice, severe neutrophil infiltration, followed by eosinophil infiltration, replaced the monocyte/macrophage infiltration. This was associated with low levels of interleukin 10 (*IL-10*) and elevated levels of *CXCL1*, *CXCL2*, *IL-1β*, and granulocyte colony-stimulating factor (*G-CSF*) in the feet of *CCR2*-KO arthritic mice [11]. While IL-10 is an anti-inflammatory factor that inhibits neutrophil recruitment [12], CXCL1 and 2 are known for their role in neutrophil migration [13], and G-CSF has been identified as a key growth factor for neutrophil development and recruitment involved in inflammatory arthritis [14]. These data suggest that multiple mediators are involved in the switch from predominantly monocyte/macrophage to neutrophil infiltrates after infection with the chikungunya virus. The replacement of monocytes with neutrophils in *CCR2*-KO mice was also observed following herpes simplex virus (HSV)-1 infection [15]. This suggests that viral infection can trigger different chemokines/receptors and pathways to compensate for the lack of *CCR2* and monocyte recruitment, thus leading to increased expression of *CXCLs*, which are known to attract neutrophils. However, in mechanical knee injuries, no compensatory mechanisms or factors have been reported yet for *CCR2* deficiency. Collectively, these data indicate the complexity of CCL2 function in cartilage.

Previous in vitro studies have shown that treatment with IL-1β or LPS increased the expression of *Mcp-1* by five- to seven-fold in chondrocytes [2]. In addition, treatment with MCP-1 inhibited chondrocyte proliferation, induced apoptosis, and increased the expression of *CCR2* (the high-affinity receptor of MCP-1) and matrix metalloproteinases (*MMP*s) 3 and 13 [19]. Furthermore, our recent in vitro studies showed that under high glucose conditions, the mRNA expression levels of *Mcp-1*, *Mmp13*, and osteocalcin (*Oc*) were significantly increased in the mouse chondrogenic cell line ATDC5. However, treatment with bindarit, a specific inhibitor of MCPs, downregulated *Mmp13* and *Oc* mRNA levels and upregulated *Col2* mRNA levels in these cells under high-glucose conditions [20]. These in vitro data indicated the importance of the MCP-1–CCR2 axis in the initiation and progression of cartilage degeneration without the intervention of inflammatory cells.

**Figure 1:**
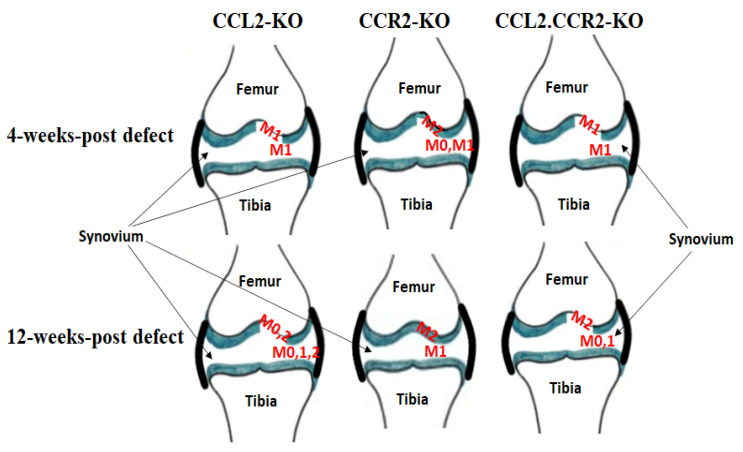
Schematic showing the most abundant macrophage population that migrated to knee joints in the 3 strains of mice after cartilage defect (summary from Jablonski et al. [21]). Articular cartilage in green.

To test whether CCL2 is also involved in the migration of mesenchymal stem cells (MSCs) to support cartilage regeneration, Jablonski et al. [21] used a mouse model of full-thickness cartilage defect (FTCD) at the knee joint. *CCL2*-KO mice showed MSCs homing to the adjacent synovium and FTCD site post-injury, but no cartilage repair was observed in these mice, suggesting that CCL2 is not necessary for the recruitment of MSCs to the FTCD site but is required for chondrogenesis (Table 1). However, *CCR2*-KO mice did not show undifferentiated MSCs in the region of FTCD but exhibited more articular cartilage regeneration at the site of FTCD compared to wild-type and *CCL2*-KO mice. In that study, macrophage evaluation revealed the presence of F4/80+, a marker of M0, CD38+, a marker of M1, and CD206+, a marker of M2, in the synovial tissue, FTCD site, and underlying subchondral bone. The numbers of M0, M1, and M2 macrophages differed between KO and wild-type mice (Figure 1), suggesting that the differences in the subpopulations of macrophages contributed to the differences in endogenous cartilage regeneration capacity between KO and wild-type mice or that factors secreted by these macrophages play a role in determining the fate of MSCs at the injured site. Indeed, it has been shown in vitro that M1 and M2 macrophages and their secreted products can regulate the chondrogenic ability of MSCs [22]. However, their effect on endogenous cartilage regeneration in vivo requires further investigation.

In summary, these findings demonstrate that MCP-1 can induce matrix-degrading enzymes responsible for cartilage degradation, but it is unclear if this effect is always mediated through binding to CCR2 because a CCR2 antagonist did not impede arthritis development in the knees of a monosodium iodoacetate (MIA)-induced rat AO model [19]. In addition to its role in cartilage degradation, in vivo studies using full-thickness cartilage defects [21] have shown that MCP-1, but not its receptor, is also required for cartilage formation (Table 2); however, the mechanism through which MCP-1 affects cartilage formation still needs to be investigated.

#### 3.1.2. MCP-2 and MCP-3

These proteins are known as CCL8 and CCL7. Both proteins bind to CCR1, CCR2, and CCR3; however, CCL8 also binds to CCR5. Recent studies have reported the involvement of these two chemokines in OA pathogenesis. *CCL7* and *8* are upregulated in the synovial fluid of patients with OA, and CCR2+ cells are abundant in the human synovium and associated with OA cartilage erosion [5]. Furthermore, one of the receptors of CCL7 and CCL8, CCR1, was found in human nucleus pulposus (NP) cells, and stimulation with IL-1β increased the expression of *CCL7* as well as cartilage-degrading enzymes, such as *MMP3* and *MMP13* [23]. However, treatment with CCL7 did not modulate the extracellular matrix or expression of cytokines or chemokines [23]. This indicated that IL-1β is the predominant regulator of cytokines and chemokines; nonetheless, CCL7 alone may not significantly affect cartilage metabolism. 

In our recent in vitro studies, we observed increased mRNA expression of *Ccl2*, *Ccl7,* and *Ccl8* in chondrogenic ATDC5 cells under high-glucose conditions (Table 2). This was associated with the increased expression of chondrocyte differentiation markers [20]. To determine whether these chemokines mediate the effect of high glucose in chondrocytes, MCP synthesis was inhibited by treatment with bindarit, which has been identified as an anti-inflammatory agent and a specific inhibitor of MCP synthesis [40]. Bindarit treatment reduced the expression of the three *Mcps* (Table 2), downregulated *Mmps* and *Oc*, and increased the expression of *Col2* mRNA in chondrocytes under high-glucose conditions. This suggests that MCPs mediate the catabolic effect of high glucose levels in chondrocytes independent of inflammatory cells. 

### 3.2. Regulated on Activation, Normal T-Cell Expressed, and Secreted (RANTES)

RANTES, also known as CCL5, binds to CCR1, CCR3, and CCR5. It acts as a chemoattractant for T cells, lymphoid cells, and stem cells. RANTES has been detected in the articular cartilage of patients with OA and rheumatoid arthritis (RA) [41]. Previous studies in patients with OA have associated high levels of *RANTES* in the synovial fluid with articular cartilage lesions [9]. In vitro studies have shown the induction of *RANTES* expression by IL-1β in chondrocytes [26] (Table 2). However, treatment with IL-4, which was downregulated in OA cartilage, attenuated the effect of IL-1β on the expression of *RANTES*, *MIP-1α*, *MIP-1β*, *MMP-13,* and a disintegrin and metalloproteinase with thrombospondin motifs 4 (*ADAMTS-4*), but did not affect the expression of growth-regulated alpha (*GRO-α*), *IL-8*, *ADAMTS-5*, or *TIMP-Metallopeptidase 1* or *3* [26]. Based on previous studies that showed a critical role of NF-κB in RANTES expression [27] and IL-4-dependent STAT6′s ability to inhibit *Rantes* gene transcription [24], we can attribute the inhibitory effect of IL-4 in OA knees to STAT6, which interferes with both NF-κB and interferon regulatory factor-1 [42,43,44,45]. 

The ability of stem cells to self-renew and their multi-lineage differentiation potential make them ideal candidates for tissue regeneration strategies. In this context, Wangler et al. [46] tested the effect of RANTES on the migration of CD146-positive (+) and CD146-negative (−) MSCs in vitro and in a degenerated organ culture model. CD146+ cells showed a high migration rate toward RANTES both in vitro and in organ culture; however, CD146− MSCs induced a greater regenerative response in resident intervertebral disc cells than CD146+ cells. Based on these data, it was proposed that both CD146-expressing and CD146− MSCs are required for intervertebral disc repair. Mechanistically, while CD146+ MSCs provided a cell source for repopulation, CD146− MSCs promoted the regenerative ability of the resident intervertebral disc cells. Furthermore, a recent in vitro study [47] has reported a strong chemotactic effect of RANTES on annulus fibrosus cells. However, when using intervertebral disc organs ex vivo or sheep cervical discs in vivo, RANTES did not stimulate the homing of annulus fibrosus cells toward the defect sites in cultured intervertebral discs under dynamic loading conditions and did not show any repair effect in vivo [47]. This suggests that RANTES can only induce chemotaxis of annulus fibrosus cells but cannot stimulate endogenous repair of the nucleus pulposus (NP). Indeed, Du et al. [28] showed that annulus fibrosus cells express CD146 marker, and treatment of annulus fibrosus with transforming growth factor β1 (TGF-β1) upregulated the expression of functional annulus fibrosus markers and increased cell contractility, indicating that TGF-β1-pre-treated annulus fibrosus cells may be an appropriate cell source for annulus fibrosus tissue regeneration [28]. In this context, Frapin et al. [48] developed a delivery system composed of pullulan microbeads that can deliver RANTES to recruit disc stem/progenitor cells and release a synergistic cocktail of transforming growth factor-beta 1 (TGF-β1) and growth differentiation factor 5 (GDF-5) to stimulate the synthesis of nucleus-pulposus-like extracellular matrices. This sequential release system significantly increased the distance that human adipose-derived stem cells (hASCs) could travel to migrate toward the nucleus pulposus in an ex vivo degenerated ovine intervertebral disc model. In addition, the overall nucleus pulposus cellularity, COL2, and aggrecan staining intensity, as well as the tyrosine receptor kinase-positive progenitor cell density in the nucleus pulposus, were increased in the treated group compared to the control group. The results of the study provided evidence that pullulan microbeads loaded with RANTES, TGF-β1, and GDF-5 constitute an innovative sequential release system for endogenous intervertebral disc regeneration [48]. This suggests that RANTES can attracts MSCs to the injured sites. Then, TGF-β1 initiates chondrogenesis of mesenchymal progenitor cells to form cartilage (Table 2), and this needs to be tested for articular cartilage defect in other OA joints.

### 3.3. Macrophage Inflammatory Proteins (MIPs) 

#### 3.3.1. MIP-1α and MIP-1β

Two major forms of MIPs in humans are MIP-1α and MIP-1β, which are also called CCL3 and CCL4, respectively. They are mostly produced by macrophages and monocytes upon stimulation by bacteria or inflammatory cytokines; however, they are also produced by several other cell types, including hematopoietic cells, fibroblasts, epithelial cells, vascular smooth muscle cells, and platelets upon activation. The plasma levels of *CCL3* and *CCL4* were associated with the severity of X-ray-defined knee OA in humans [49]. Regarding the molecular pathways involved in the regulation of these two chemokines (Table 2), resistin, an adipokine, is involved in promoting the expression of these two chemokines and was found to be elevated in patients with rheumatoid arthritis (RA) and OA, as well as in synovial joints after injury [50,51,52,53]. Resistin stimulates several chemokines in human articular chondrocytes (Table 2), which are known to play important roles in inflammatory diseases, such as RA and OA [30,54]. In this respect, Zhang et al. [30,54] demonstrated that resistin stimulates the expression of *MIP-1α* and *MIP-1β* in human articular chondrocytes through the coregulation of CCAAT enhancer binding protein beta (C/EBPβ) and NF-κB. Furthermore, recent studies have identified two miRNAs that regulate the expression of *Mip-1α*: miR-122 and miR-451 [25]. They directly target the *Mip-1α* gene by binding to its 3′UTR. Both miR-122 and miR-451, which were found to be upregulated in OA articular cartilage, affected the expression of *MIP-1α* and *RANTES* in stimulated rat chondrocytes (Table 2) [25]. While miR-122 reduced the stimulatory effect of *IL-1β* on *IL-1α*, *IL-2*, *IL-4*, *IL-6*, *GM-CSF*, *MIP-1α*, *RANTES,* and VEGF [25], miR-451 increased the expression of *IL-2*, *IL-4*, IL-6, GM-CSF, and *MIP-1α* and reduced *VEGF* expression [25]. 

Yang et al. [31] identified one miRNA that regulated the expression of *Mip-1β,* because reduced expression of *Mip-1β* in the articular cartilage of MMD-induced OA joints was associated with increased levels of miR-495 and reduced expression of *COL2* and *NF-κB* compared to control joints [31]. Moreover, chondrocytes from OA articular cartilage transfected with miR-495 mimic exhibited reduced expression of *MIP-1β*, *p65*, *p50*, and IκBα phosphorylation, and transfection with *Mip-1β* siRNA reduced the expression of *MIP-1β*, *p65*, *p50,* and IκBα phosphorylation in chondrocyte cells. However, inhibition of miR-495 increased the expression of *MIP-1β*, *p65*, *p50*, and IκBα phosphorylation and accelerated chondrocyte proliferation accompanied by reduced apoptosis in the chondrocytes of mice with OA [31]. These results demonstrated that inhibition of miR-495 suppressed chondrocyte apoptosis and promoted its proliferation through activation of the NF-κB signaling pathway and upregulation of *MIP-1β* in OA, suggesting the involvement of MIP-1β in cartilage regeneration.

These findings demonstrate that miRNAs are involved in articular cartilage degeneration and regeneration by controlling chemokine expression. However, further in vivo studies are required to reveal the crosstalk between miRNAs and chemokines in cartilage pathology and chondrogenesis (Table 2).

#### 3.3.2. MIP-3

Two chemokines have been identified thus far in the MIP-3 group: MIP-3α (CCL20) and MIP-3β (CCL19) [3]. CCL20 binds to CCR6 [16], and CCL19 functions by binding to CCR7. Both chemokines and their receptors are upregulated in OA knees compared with healthy knees. 

**MIP-3β/CCL19:** The expression of CCL19 in the synovial tissue is associated with knee-related difficulty in daily activities independent of other OA factors, and the expression of its receptor CCR7 is significantly associated with the severity of OA symptoms in both humans [55] and animals [17]. In addition, *CCR7*-deficient mice showed delayed cartilage degeneration in MMD knees from *CCR7*-KO mice compared to wild-type mice [17]. These findings suggest that CCL19, or another chemokine ligand, binds to CCR7 to induce cartilage degeneration. 

**MIP-3α/CCL20:** Alaaeddine et al. [32] reported increased expression of *CCL20* and its receptor, *CCR6,* in the cartilage of patients with OA knees compared with healthy individuals. Furthermore, treatment of healthy cartilage explants with CCL20 ex vivo stimulated the secretion of matrix-degrading enzymes and proteoglycans, increased the expression of collagen type X (*COL10*), and inhibited *COL2* [32], suggesting an important role of CCL20-CCR6 in the pathogenesis of cartilage.

### 3.4. CCL21 

CCL21 is another chemokine that binds to CCR7. CCL21 controls the migration of monocytes/macrophages, mature dendritic cells (DCs), and naïve T cells to lymph nodes [56,57,58,59]. Previous studies have reported elevated mRNA expression of *Ccl21* in the joints of both young and old mice, in which injury was experimentally induced through MMD [60,61,62]. *CCL21* was localized in chondrocytes, meniscal cells, and the growth plate matrix. The levels of *CCL21* were also increased in the MMD joints of young mice and old sham control joints. Furthermore, the results of our recent studies [33] using MMD to induce OA in Sprague–Dawley adult rats showed that treatment of MMD knees with CCL21 neutralizing antibodies altered the mRNA expression of matrix-degrading enzymes and mitigated post-MMD inflammation, as evidenced by a reduction in the expression of *Il-6* and *Mmps* as well as a reduction in inflammatory cell infiltrates in the joints of MMD-operated knees early post-MMD [33]. Overall, these data suggested that the expression of cartilage-degrading enzymes depends on *Ccl21* expression. This is likely due to the chemotactic effect of CCL21 on inflammatory cells that secrete matrix-degrading enzymes. Therefore, the alteration of CCL21′s function may limit cell infiltration and consequently reduce cartilage degradation. Moreover, a previous study by Sambamurthy et al. [17] showed that the lack of the CCL21 high-affinity receptor, CCR7, in *CCR7*-KO mice resulted in protection from early cartilage degeneration and osteophyte formation post-MMD compared to control, wild-type mice. 

To investigate the role of the CCL21–CCR7 axis in cartilage repair, Joutoku et al. used a mouse model of articular cartilage with full-thickness defect [18] in juvenile and adult *CCR7*-KO and wild-type mice. In that study, the lack of *CCR7* in juvenile mice significantly reduced the capacity for cartilage repair, whereas *CCR7*-KO adult mice exhibited identical repair with scar-like tissue to wild-type mice. Additionally, CCL21 was transiently expressed around the damaged cartilage in juvenile mice, whereas in adult mice, it was mainly expressed in the bone marrow and rarely at the injury site [18]. These data demonstrate that the CCL21–CCR7 axis may not affect cartilage healing in adult mice because of the absence of CCL21 expression in the injured areas. In vitro evaluation using MSCs isolated from wild-type and *CCR7*-KO mice revealed that *CCL21* significantly increased MSC migration in wild-type mice, but not in *CCR7*-KO mice, confirming that CCL21 requires the CCR7 receptor to regulate MSC migration [18]. Therefore, to evaluate the effect of CCL21 on MSC migration in vivo, osteochondral defects were induced in 16-week-old female, Japanese, white rabbits. Local administration of exogenous CCL21 decreased scar-forming healing and enhanced hyaline cartilage repair in vivo. Together, these data indicate that the CCL21–CCR7 axis may play a role in the molecular pathways that control both cartilage repair and degeneration and suggest that the local molecular environment may direct the CCL21–CCR7 axis toward anabolic or catabolic functions.

### 3.5. CCL22

CCL22 was named the macrophage-derived chemokine (MDC) because it was initially isolated from human monocyte-derived macrophages [63]. CCL22 binds with high affinity to CCR4, a G-protein-coupled receptor, and induces calcium mobilization in CCR4-expressing cells [64]. CCR4 is expressed in various T cell subsets and myeloid cells [65] and binds to CCL17 and CCL22 with high affinity. In addition to macrophages and dendritic cells, chondrocytes present in OA cartilage (human and rat models) co-express CCL22 and cleaved caspase-3 (a marker of apoptosis) [66], suggesting that, in addition to regulating chemotaxis, in part through calcium signaling, CCL22 may also regulate pathways that lead to cartilage degeneration. Indeed, Ren et al. [66] demonstrated that CCL22 induces apoptosis in isolated human chondrocytes. Furthermore, Xu et al. [34] showed that CCL22 knockdown reduced the expression of its specific receptor *CCR4*, other pro-inflammatory cytokines, and matrix-degrading enzymes and improved the viability of LPS-stimulated ATDC5 cells. However, CCR4 overexpression rescued the expression of pro-inflammatory effectors and decreased cell viability [34]. These data demonstrate the importance of the CCL22–CCR4 axis in cartilage degeneration, which needs to be tested in vivo.

### 3.6. ELR+ CXC Chemokines

ELR+ CXC, such as CXCL5 and CXCL6, are small chemotactic cytokines belonging to the CXC family. They are characterized by the presence of a glutamic acid–leucine–arginine (ELR+) motif preceding the CXC sequence. They are potent angiogenic chemokines that induce endothelial chemotaxis and neovascularization [67]. CXCL5 binds to CXCR2, whereas CXCL6 binds to both CXCR1 and CXCR2. CXCL6, also known as granulocyte chemotactic protein-2 (GCP2), acts as a chemoattractant for neutrophilic granulocytes. CXCL6 is involved in several processes, such as inflammation, cell growth, and metastasis, through its interaction with the chemokine receptors CXCR1 and CXCR2. Previous studies [68,69] have reported the expression of CXCL6 and its receptors, CXCR1 and CXCR2, during embryonic development and in healthy adult cartilage. Furthermore, CXCR2 knockout mice exhibited more severe OA than wild-type mice after MMD [68], suggesting that CXCR2 protects the cartilage by promoting phenotypic homeostasis in articular cartilage [68]. Moreover, Kawata et al. [36] demonstrated that MSC-derived extracellular vesicles upregulated the expression of CXCL5 and CXCL6 in chondrocytes, and, when injected into knee joints, they accelerated meniscus regeneration. This suggests that MSC-derived extracellular vesicles act via the CXCL5 and CXCL6/CXCR2 axes to repair cartilage damage. Indeed, recent studies have shown that blocking the function of CXCL6 in human articular chondrocytes resulted in glycosaminoglycan (GAG) loss, while treatment of the chondrogenic mouse cell line C3H10T½ with recombinant CXCL6 increased the GAG content and AKT phosphorylation and upregulated the mRNA expression of *Aggrecan* (*Acan*), which is a major proteoglycan in the cartilage, suggesting an anabolic function of CXCL6. To test the anabolic effect of CXCL6 on the cartilage in vivo, Caxaria et al. [69] used a previously validated strategy based on co-implantation of human articular chondrocytes with growth-arrested, CXCL6-overexpressing COS7 cells or green fluorescent protein-overexpressing (GFP) COS7 cells as controls [70]. Implantation was performed ectopically in the immunodeficient mice. Two weeks after implantation, CXCL6-overexpressing implants displayed enhanced ECM formation and increased the expression of *COL2* compared to GFP-expressing control cells [69]. To evaluate the role of CXCL6 in OA development, Caxaria et al. [69] generated a CXCL6-triple mutant (T) by changing the conserved lysine amino acids at positions K100E, K101E, and K105E. This triple mutation disrupted the binding of CXCL6 to GAGs on the endothelial surface, thereby inhibiting the chemotactic effects of CXCL6 on neutrophils. However, the triple mutation did not affect the capacity to activate its receptors in chondrocytes, thus retaining its chondrogenic activity. Endothelial cells display GAGs on their surfaces, to which ELR+ CXC chemokines bind, thus creating a haptotactic gradient [71,72]. Therefore, neutrophils displaying chemokine receptors interact with ELR+ CXC chemokines and are activated by chemokines immobilized on the endothelial surface, which initiate transendothelial migration [73,74,75,76]. However, the triple mutant showed a significant reduction in chemotaxis and transendothelial migration of leukocytes but was still able to induce AKT phosphorylation in chondrocytes and promote chondrogenesis [69]. The triple mutants were tested using an OA mouse model. Mice received intra-articular injections of adenoviruses encoding wild-type CXCL6, CXCL6-T, or GFP as the control after MMD. While both wild-type CXCL6 and CXCL6-T induced AKT phosphorylation, only wild-type CXCL6 induced the accumulation of neutrophils within the joint space, and CXCL6-T significantly reduced cartilage damage and osteophyte formation compared to wild-type and GFP mice. Therefore, it was speculated that CXCL6-T can be used as a disease-modifying drug for OA.

As miRNAs expressed in MSCs have been shown to regulate different mechanisms, including cell proliferation, differentiation, senescence, and survival [77,78,79,80], a recent study by Liu et al. [35] focused on the role of miRNAs in mediating the effects of MSCs. Using MSCs from healthy subjects and patients with ankylosing spondylitis (AS), they found that AS-MSCs exhibited a stronger ability to inhibit osteoclastogenesis than MSCs from healthy subjects. This effect was reversed by reducing the expression of *CXCL5*. Bioinformatics analysis and luciferase reporter assays revealed an interaction between miR-4284 and CXCL5. Moreover, in vitro studies by Liu et al. [35] showed that exogenous CXCL5 inhibits osteoclastogenesis in CD14+ cells by reducing p65 phosphorylation without affecting the JNK, p38, AKT, or extracellular regulated protein kinase (ERK) pathways. This suggests that miR-4284, which is downregulated in AS-MSCs, promotes *CXCL5* secretion from AS-MSCs, leading to inhibition of osteoclastogenesis. Collectively, these data emphasize the role of miRNAs in controlling the expression of *CXCL5*. Because osteoclastogenesis plays an important role in cartilage degeneration, CXCL5 may be a good candidate for cartilage regeneration [36]. Therefore, further investigation is warranted into the regulatory effect of miR-4284 on the expression of *CXCL5* in chondrocytes and the mechanism underlying the effect of CXCL5 on cartilage regeneration. 

### 3.7. CXCL12

CXCL12 belongs to the ELR-negative CXC subfamily and is also known as stromal-cell-derived factor-1 (SDF-1). Six isoforms have been identified (α, β, γ, δ, ε, and θ isoforms), and SDF-1α and SDF-1β are the most abundant. All isoforms were derived from *CXCL12* via alternative splicing [81,82]. CXCL12 is the only CXC chemokine with alternative splicing variants, and it is regulated and processed more at the post-translational level than at the transcriptional level. The six isoforms only differ in the length of the last exon, with the amino acid (AA) sequence determining the specific length for each isoform, including 89 AAs for α-isoform, 93 AAs for β-isoform, 119 AAs for γ-isoform, 140 AAs for δ-isoform, 90 AAs for ε-isoform, and 100 AAs for θ-isoform [82,83,84]. Most studies regarding CXCL12 focus only on the α isoform or do not distinguish among isoforms. CXCL12 can stimulate several downstream signaling pathways that regulate chemotaxis, gene transcription, cell survival, and proliferation by binding to its cognate receptors CXCR4 and ACKR3 [85,86]. CXCL12 has been classified as a homeostatic chemokine and is involved in several physiological processes, such as embryogenesis, angiogenesis, hematopoiesis, and tissue regeneration [87,88]. However, it is also involved in the pathogenesis of different diseases, such as tumor metastasis [89] and arthritis [83]. CXCL12 plays an important role in stem cell migration and proliferation and mediates the homing of stem cells to the bone marrow by binding to CXCR4 on circulating cells. Many cell types, including osteoblasts, fibroblasts, and endothelial cells, express CXCL12. By binding to its receptors, CXCL12 activates multiple signaling pathways, such as focal adhesion kinase (FAK), phosphatidylinositol-3-kinase (PI3K) [90], mitogen-activated protein kinase (MAPK), ERK, and Janus kinase tyrosine kinase 2 (Jak/Tyk2), which are involved in cell adhesion and migration [91]. The roles of CXCL12 and CXCR4 in osteogenesis are well-established [92,93,94]. However, their roles in chondrogenesis and cartilage pathogenesis have not been thoroughly investigated. In this review, we discuss recent findings regarding CXCL12/CXCR4 and cartilage.

CXCL12 and its receptors are upregulated in synoviocytes and articular cartilage with severe proteoglycan loss [37]. In vitro studies using primary chondrocytes collected from mice embryos showed strong induction of p65 and phosphorylation of IκBα, ERK, JNK, and p38 after treatment with CXC12, demonstrating that CXCL12 induces NF-κB activation in chondrocytes [37]. In addition to these pathways, CXCL12 was also found to activate the Wnt/β-catenin canonical pathway. Because it is known that activation of the NF-κB, MAPK, and Wnt/β-catenin pathways plays a vital role in aggrecanase-mediated OA [95,96,97], this suggests the involvement of the CXCL12–CXCR4 axis in cartilage degeneration.

Recent in vitro studies by Wang et al. [38] using synovium-derived stem cells showed that the CXCL12–CXCR4 axis is involved in synovium-derived stem cell migration through the PI3K/protein kinase B pathway. Furthermore, treatment with TGF-β1 induced chondrogenesis through ERK activation, and the addition of CXCL12 enhanced TGF-β1-induced chondrogenesis in synovium-derived stem cells in vitro. This effect was related to JNK activation [38]. Moreover, in vivo studies by Lu et al. [29] showed the synergistic action of CXCL12 and CCL5 in attracting bone marrow mesenchymal stem cells (BMSCs) for cartilage regeneration in a mouse model of temporomandibular joint OA. These data demonstrate that in the presence of TGF-β1, CXCL12 can enhance cell migration and chondrogenesis through the MAPK pathway. 

### 3.8. CX3CL1

In CX3CL1, also known as neurotactin or fractalkine, two cysteine residues are separated by three amino acids [4]. CX3CL1 is a membrane-bound chemokine with a chemokine–mucin hybrid structure and a transmembrane domain [98]. Its unique structure allows it to function as an adhesion chemokine when bound to the membrane and as a chemoattractant in the soluble form. Transformation from the bound to soluble form is induced via cleavage of CX3CL1 by ADAM-10 or ADAM-17 [99]. CX3CL1 promotes the extravasation of circulating leukocytes by binding to its receptor, CX3CR1, through integrin-dependent and integrin-independent mechanisms [100]. Synovial fluid and serum CX3CL1 concentrations were higher in patients with RA than in patients with OA or healthy controls [101]. A recent study by Hoshino-Negishi et al. [39] using a CX3CL1-neutralizing antibody in a mouse model of inflammatory arthritis showed a significant reduction in the plasma levels of cartilage oligomeric matrix proteins and matrix metalloproteinase 3. Neutralization of CX3CL1 blocks the migration of bone marrow osteoclast precursors, thereby inhibiting synovitis and decreasing arthritis scores by reducing cartilage and bone damage. This suggests that CX3CL1 indirectly affects cartilage through its chemotactic effect on osteoclast precursors that express the CX3CL1 receptor, CX3CR1, and that can degrade cartilage.

## 4. Conclusions

In summary, chemokines clearly influence cartilage and OA development in both positive and negative ways and can influence the development of structural diseases and symptoms. Their induction and involvement in either process depend on injury-inducing factors and the local molecular environment, and they are both site- and time-dependent. Therefore, future studies should focus on the characterization of circulating cells, local cell populations, and gene expression patterns at the injured site to determine appropriate targets for cartilage regeneration. This can be achieved using certain techniques, such as multicolor flow cytometry and single-cell RNA sequencing.

One of the challenges in defining the role of chemokines in cartilage pathology or regeneration is the apparent redundancy in the interaction of chemokines with their receptors. Because many chemokines bind multiple receptors, and multiple receptors can bind many chemokines within the same subfamily, each chemokine can potentially induce distinct signaling mechanisms depending on the receptor with which it interacts during a specific time and in a specific environment. Hence, it is crucial to determine whether targeting specific chemokines or their receptors in each situation will help develop effective therapeutic strategies for cartilage repair. However, systemic treatment for cartilage repair may cause more harm than benefit because chemokines and their receptors are expressed in multiple cell types and are involved in homeostasis.

## Figures and Tables

**Table 1:** Cartilage phenotype in chemokine or chemokine-receptor-deficient mice.

Chemokine/Receptor		KO Phenotype		References
Chemokine Name	Specific Receptors *	Chemokine	Receptor	
MCP-1/CCL2	CCR2	Protected from cartilage degradation post-MMD. No cartilage regeneration in FTCD.	Protected from cartilage degradation post-MMD. Enhanced cartilage regeneration in FTCD. Aggravated cartilage degradation post-CHIKV infection.	* [16] [5,11] [11]
MIP-3β/CCL19	CCR7	-	Delayed cartilage degeneration post-MMD.	* [16] [17]
CCL21	CCR7	-	Delayed cartilage degradation post-MMD. Reduced cartilage repair in FTCD in juvenile, but no change in adult mice.	* [16] [17] [18]

* Reference for CC-CCR. CHIKV: Chikungunya virus. FTCD: Full-thickness cartilage defect. MMD: Medial meniscus destabilization. -: undetermined.

**Table 2:** Factors that regulate the expression or synthesis of chemokines and the possible involvement of chemokines in cartilage degradation and regeneration based on in vitro and in vivo studies.

Chemokines	Regulators of Chemokine Expression/Synthesis		Role		References
Chemokine Name	Inhibitors	Stimulators	Degradation	Formation	
MCP-1/CCL2	Bindarit	Il-1β, LPS High glucose	Yes	Yes	[20]
					[2]
					[19]
					[21]
MCP2/CCL8	Bindarit	High glucose	-	-	[20]
MCP3/CCL7	Bindarit	High glucose Il-1β	-	-	[20]
					[23]
RANTES/CCL5	Il-4, STAT6 miR-122	Il-1β NF-_k_B	-	CCL5 + TGF-β1 CCL5 + CXCL12	[24]
					[25]
					[26]
					[27]
					[28]
					[29]
MIP-1α/CCL3	miR-122	Resistin, Il-1β miR-451	Yes	-	[25]
					[30]
MIP-1β/CCL4	miR-495	Resistin Il-1β	-	Yes	[31]
					[30]
MIP-3α/CCL20	-	-	CCL20	-	[32]
CCL21	-	-	CCL21-CCR7 in MMD	CCL21-CCR7 in FTCD in juvenile	[33]
					[17]
					[18]
CCL22	-	-	Yes	-	[34]
CXCL5	miR-4284	MSC-DEV	-	CXCL5/CXCL6-CXCR2	[35]
					[36]
CXCL6/GCP2	MSC-DEV	-	-	CXCL5/CXCL6-CXCR2	[36]
CXCL12	-	-	CXCL12	CXCL12 + CCL5 CXCL12 + TGF-β1	[37]
					[29]
					[38]
CX3CL1/Fractalin	-	-	CX3CL1	-	[39]

MSC-DEV: Mesenchymal-stem-cell-derived extracellular vesicles. FTCD: Full-thickness cartilage defect. MMD: Medial meniscus destabilization. -: Undetermined.

## Data Availability

Not applicable.
